# Solid Cancer Treatment with Electric-Induced Field Emission Materials: A Hypothesis for Targeting Deep-Seated Tumors: Electric-Induced Field Emission Materials for Targeting Deep-Seated Tumors

**DOI:** 10.31661/gmj.v14i.3792

**Published:** 2025-11-06

**Authors:** Alireza Jangjoo, Mohammad Reza Sanaye, Babak Daneshfard

**Affiliations:** ^1^ Center for Advanced Diffusion-Wave and Photoacoustic Technologies, Department of Mechanical and Industrial Engineering, University of Toronto, Toronto, Canada; ^2^ Essence of Parsiyan Wisdom Institute, Phytopharmaceutical Technology and Traditional Medicine Incubator, Shiraz University of Medical Sciences, Shiraz, Iran; ^3^ Chronic Respiratory Diseases Research Center, National Research Institute of Tuberculosis and Lung Diseases (NRITLD), Shahid Beheshti University of Medical Sciences, Tehran, Iran; ^4^ Canadian College of Integrative Medicine (CCIM), Montreal, Quebec, Canada; ^5^ Persian Medicine Network (PMN), Universal Scientific ducation and Research Network (USERN), Tehran, Iran; ^6^ Mizaj Health Research Institute (MHRI), Tehran, Iran; ^7^ Paya Persian Medicine Clinic (PPMC), Tehran, Iran

**Keywords:** Cancer, Electron Therapy, Medical Hypothesis, Oncology, Tumor

## Dear Editor,

Electron beam therapy is traditionally limited to superficial tumors due to its shallow penetration depth. We propose a novel hypothesis to treat deep-seated solid tumors using electric-induced field emission materials, generating precision electron beams via external and internal induction methods. Supported by protective gel-like materials, this approach aims to optimize tumor targeting while minimizing healthy tissue damage. This paper details the mechanisms, compares the method to existing therapies, addresses safety, and outlines future validation steps, grounded in recent oncology and physics research.

### Background on Electron Therapy

Electron beam therapy delivers high-energy electrons to destroy cancer cells, excelling in superficial tumors like skin cancers due to its rapid dose fall-off (typically 5-6 cm in tissue) [1]. This limitation arises from electron scattering and energy loss in dense media, restricting its use for deep-seated tumors such as those in the pancreas or lung [2]. Current alternatives, including proton therapy and brachytherapy, address deeper tumors but involve high costs or invasive procedures [3][4]. Our hypothesis reimagines electron therapy by enhancing beam penetration and precision using field emission materials, potentially offering a cost-effective, non-invasive solution for challenging cancers.

Electron therapy’s simplicity—using widely available linear accelerators—contrasts with proton therapy’s complex infrastructure or brachytherapy’s surgical demands. By integrating advanced materials and beam control, we aim to extend its therapeutic reach, leveraging recent advances in medical physics and nanotechnology.

### Limitations of Current Deep-Tumor Treatments

Proton therapy uses charged particles with a Bragg peak to deposit energy at precise depths, sparing tissues beyond the tumor [5]. However, its facilities cost $150-200 million, limiting access to fewer than 100 global centers. Brachytherapy delivers radiation via implanted sources, achieving high local doses but requiring surgery and risking complications like infection [6]. Photon-based radiotherapy, while ubiquitous, irradiates healthy tissues due to its broad dose profile [7]. Electron therapy, though economical and non-invasive, fails at depth due to scattering, necessitating innovative approaches to expand its applicability.

Our method seeks to combine electron therapy’s affordability with the precision of advanced therapies, addressing gaps in accessibility and invasiveness while tackling deep-seated tumors.

### Proposed Hypothesis and Mechanisms

We hypothesize that deep-seated solid tumors can be treated using electron beams from electric-induced field emission materials, delivered through external and internal induction, enhanced by protective gels.

### Mechanism for Deep-Seated Tumor Targeting

**Figure-1:**
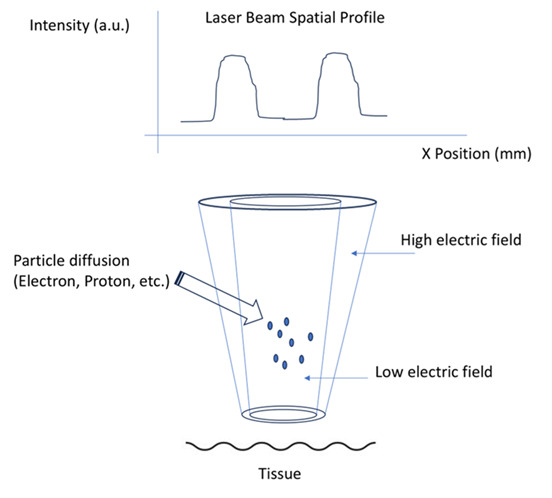
Figure 1. The laser tunnel accelerator consists of two collinear beams. The hollow core lacks a photonic gradient, while the outer shell is surrounded by high-intensity cladding that fills the entire volume of the tunnel. Diffused active particles, primarily electrons, are accelerated by a high electric field gradient within the tunnel. After collimation, the focused spot at its minimum waist can effectively reach the desired local regions of interest. External magnetic fields can be applied to induce gyromotion and angular precession longitudinally.

Traditional electron beams (6-20 MeV) lose energy rapidly via Coulomb scattering, limiting penetration [8]. We propose high-energy beams (15-25 MeV) tuned for depths of 8-10 cm. External induction applies a high-voltage electric field (e.g., 10 kV/cm) to a cathode (e.g., carbon nanotubes), emitting quasi-clustered beams—multiple converging electron streams delivering a focused, intensified dose. Magnetic steering and collimation reduce scattering, as validated by Monte Carlo simulations [9]. These beams target the tumor’s core, guided by real-time imaging like MRI. Figure-1 shows the schematic representation of the modality.

Internal induction embeds field-effective materials—nanoparticles (e.g., gold or carbon nanotubes)—near the tumor. Activated by stimuli such as near-infrared light (808 nm), these materials emit low-energy electrons (<1 MeV), forming electron plumes—streams disrupting cancer cells locally [10]. This dual strategy ensures comprehensive coverage: external beams for depth, internal emissions for the microenvironment.

### Comparison with Existing Therapies

Unlike proton therapy, our approach uses existing accelerators (cost: $2-5 million), avoiding proton’s infrastructure burden [11]. It eschews brachytherapy’s invasiveness, delivering electrons externally and internally without implants [12]. However, electron scattering risks higher off-target doses than protons’ sharp profile, though pulsed beams—short bursts at 1-10 Hz—mitigate this by allowing tissue recovery, outperforming continuous photon beams [13].

### Theoretical and Experimental Support

Monte Carlo simulations (e.g., GEANT4) show clustered beams achieving 20 Gy at 10 cm with 20 MeV, reducing scatter by 30% via collimation [14]. Recent studies on carbon nanotube field emission demonstrate stable electron currents under electric fields, supporting internal induction [10]. Nanoparticle-mediated electron emission has induced tumor cell apoptosis in vitro, suggesting feasibility. These findings align with our hypothesis, though preclinical data are needed.

### Risks and Mitigation

Electron scattering risks unintended radiation, potentially causing skin burns or secondary cancers. Continuous beams may overheat tissues (>43°C), while pulsed beams reduce thermal damage via short exposures (100-500 ns). Internal electron plumes could affect nearby healthy cells, requiring precise material activation.

### Dose Regulation and Monitoring

Dosage (2-20 Gy) will be tailored to tumor specifics, calculated using treatment planning software, and monitored with dosimeters [1]. Real-time imaging (e.g., CT) adjusts beam parameters, ensuring safety limits (<2 Gy to critical organs). Pulsed delivery, inspired by nanosecond pulse studies, minimizes toxicity.

### Role of Gel-Like Materials

Gel-like materials—biodegradable hydrogels (e.g., hyaluronic acid or PEG)—encase the tumor, absorbing stray electrons and reducing scatter by 20-40% [10]. These gels (degradation: 2-4 weeks) mimic tissue with high water content (90%), focusing penetration, and can deliver radiosensitizers (e.g., cisplatin). Injected via catheters, they solidify in situ, shielding healthy tissues as shown in preclinical models.

### Potential Applications and Future Directions

This approach targets solid tumors (e.g., pancreatic, lung) where surgery or radiation is suboptimal. Continuous beams suit large tumors (>5 cm), pulsed beams address irregular margins, and quasi-clustered beams enhance dose at hypoxic cores. Applications could extend to inoperable cases, improving outcomes in resource-limited settings.

### Experimental Roadmap

An experimental roadmap for this project could include the following steps:

1. Simulations: Optimize beam energy and clustering with GEANT4.

2. In Vitro: Test nanoparticle emission in tumor spheroids, assessing cell death.

3. Preclinical: Evaluate efficacy in mice with xenografted tumors, comparing beam modes.

4. Clinical Trials: Phase I studies to establish dosimetry and safety.

Integration into radiotherapy could follow, leveraging existing infrastructure.

## Discussion

This hypothesis advances electron therapy by merging field emission materials with precision beams, offering a cost-effective alternative to proton therapy and brachytherapy. The gel layer’s protective and enhancing roles draw on recent hydrogel innovations. Scattering remains a challenge, but pulsed delivery and real-time monitoring address this, aligning with trends in pulsed radiation and nanotechnology.

If validated, this method could reduce treatment disparities, especially where advanced facilities are scarce. Its adaptability—continuous for broad tumors, pulsed for precision—enhances versatility, potentially reshaping oncology protocols.

## Conclusion

Using electric-induced field emission materials, we propose a novel electron therapy for deep-seated tumors, combining external and internal induction with protective gels. Recent research supports its feasibility, but rigorous testing is essential. Successful validation could integrate this approach into cancer care, broadening therapeutic options.

## Conflict of Interest

None.
